# Smaller prize, bigger size? Exploring the impact of money on men’s self-reported markers of masculinity

**DOI:** 10.3389/fpsyg.2023.1105423

**Published:** 2023-02-01

**Authors:** Jacob Dalgaard Christensen, Tobias Otterbring, Carl-Johan Lagerkvist

**Affiliations:** ^1^Department of Economics, Swedish University of Agricultural Sciences, Uppsala, Sweden; ^2^Department of Management, University of Agder, Kristiansand, Norway

**Keywords:** penis size, monetary rewards, self-report, data quality, masculinity, bodily cues, online surveys

## Abstract

Bodily markers, often self-reported, are frequently used in research to predict a variety of outcomes. The present study examined whether men, at the aggregate level, would overestimate certain bodily markers linked to masculinity, and if so, to what extent. Furthermore, the study explored whether the amount of monetary rewards distributed to male participants would influence the obtained data quality. Men from two participant pools were asked to self-report a series of bodily measures. All self-report measures except weight were consistently found to be above the population mean (height and penis size) or the scale midpoint (athleticism). Additionally, the participant pool that received the lower (vs. higher) monetary reward showed a particularly powerful deviation from the population mean in penis size and were significantly more likely to report their erect and flaccid penis size to be larger than the claimed but not verified world record of 34 cm. These findings indicate that studies relying on men’s self-reported measures of certain body parts should be interpreted with great caution, but that higher monetary rewards seem to improve data quality slightly for such measures.

*“Men read maps better than women because only men can understand the concept of an inch equaling a hundred miles.”* – Roseanne Barr

## 1. Introduction

Men’s physical attributes, such as their height, body type, and penis size, have been shown to predict a wide array of phenomena, ranging from body satisfaction, self-view, and feelings of masculinity (Grogan and Richards, 2002; Hall, 2006; Lever et al., 2006) to mating success, income levels, and consumption preferences (Judge and Cable, 2004; Puts, 2010; Otterbring et al., 2020; Richardson et al., 2023). In fact, New Guinean Yupno men even refer to the penis for number 33 in the unique Yupno body count system (Wassmann and Dasen, 1994; Kramer, 2022).

While no clear consensus seems to exist regarding the relationship between men’s penis size and women’s preferences (Štulhofer, 2006; Mautz et al., 2013; Prause et al., 2015), or their sexual satisfaction (Eisenman, 2001), it is arguably fairly well-established that this particular factor constitutes a conspicuous marker of masculinity (Lehman, 1998; Lever et al., 2006; Ostberg, 2010). Indeed, although Freud’s (1925) penis envy concept was meant to psychoanalytically proclaim a female envy for the male reproductive organ, some studies have found symbolic support for penis envy even in men (Hall and Van de Castle, 1965; Melnick, 1997; Gottlieb, 2004; Domhoff, 2013), with many men being concerned about their penis size, supporting the notion that size seems to matter (Lever et al., 2006; Johnston et al., 2014). For example, one study on more than 50,000 heterosexual men and women found that only 55% of men were satisfied with their penis size, although 85% of women expressed satisfaction with their partner’s penis size (Lever et al., 2006). Nevertheless, a more recent study found that 20% of women reported having ended a relationship partly because their partner’s penis size was too small for their personal preferences (Prause et al., 2015). Moreover, Lever et al. (2006) found that almost 9 of 10 men self-reported their penis as either average or large, while only 12% of men reported it to be small. Despite these estimates, 45% of men still wanted their penis to be larger, while only 0.2% preferred a smaller penis.

Beyond penis size, height as well as other bodily markers that signal physical dominance have been shown to be important for men’s reproductive (Pawlowski et al., 2000; Nettle, 2002), occupational (Judge and Cable, 2004; Case and Paxson, 2008), and financial success (Deaton and Arora, 2009; Tyrrell et al., 2016). Accordingly, many men exaggerate their height and athleticism on online dating sites to boost their chances on the mating market (Ellison et al., 2006, 2012; Toma et al., 2008; Toma and Hancock, 2010; Burke and Carman, 2017). One plausible reason for men’s desire to be tall, and for women’s preference for men with an imposing stature and other formidability features, is the link between male stature and status (Jackson and Ervin, 1992; Buss, 2016; Otterbring et al., 2018), which appears to be more than metaphorical, considering that we tend to “look up to” tall individuals, as evidenced from their many benefits in life (Schubert, 2005; Stulp et al., 2013). In other words, just as the “what is beautiful is good”-stereotype (Dion et al., 1972) postulates that physically attractive individuals are evaluated far more favorably even on traits and characteristics that have nothing to do with their looks, people also hold a “height halo,” in which tall people are portrayed and perceived more positively as a function of their “altitude advantage.” Similarly, sometimes weight signals importance (Jostmann et al., 2009), suggesting that men may over-report their weight to signal masculinity, power, and potency (Roberts, 1995; Ambwani and Chmielewski, 2013; Devia et al., 2021).

The results delineated above indicate that men may exaggerate their penis size, height, and presumably other factors linked to their physique in self-report situations, partly because men and, to some extent, women seem to equate bigger with better when it comes to male markers of masculinity (Frederick and Haselton, 2007; Mautz et al., 2013; Johnston et al., 2014). Therefore, the aim of this study was to examine whether men at the aggregate level would overestimate their height, weight, athleticism, and penis size in a self-report study, and if so, to what extent. Admittedly, people show a general propensity to present themselves in an ego-boosting way, as self-serving tendencies captured by, for example, the better-than-average effect, the positivity bias, and the optimism bias help defending, maintaining, and enhancing a favorable self-view (Mezulis et al., 2004; Paulhus, 2017; Otterbring and Mitkidis, 2018; Zell et al., 2020). Notwithstanding the almost universal human tendency to exaggerate things in a self-serving way, few studies have compared the magnitude of such exaggerations in the same study across a series of related but still distinct measures. In that sense, the current work contributes to the literature not so much regarding *if* participants would exaggerate certain male markers of masculinity but rather *the magnitude* of this exaggeration.

As a second main objective, the study sought to explore whether the amount of monetary rewards could reduce the extent of such potentially exaggerated responses. The latter aim is relevant in light of evidence linking increased monetary compensation to improved data quality under certain circumstances (Litman et al., 2015; Balasubramanian et al., 2017). Given that a growing body of research relies on self-reported online surveys, it is important to understand which implications such data collection techniques have for scholars’ ability to draw reasonable conclusions.

Together, the present research contributes to the literature in two crucial ways. First, our findings reveal that self-report data related to men’s bodily markers of masculinity cannot necessarily be trusted and, as such, should be interpreted with great caution, as men generally exaggerate their size on such measures. Second, however, these exaggerated responses seem to be at least partially contingent on the amount of monetary compensation given to participants, and may, therefore, be meaningfully mitigated by larger (vs. smaller) payments.

## 2. Methods

The data were collected in connection to another project, which included a large number of measures (approximately 200 items) on consumption preferences, life history traits (e.g., birth order, number of siblings, parental status, number of offspring desired), and subjective life expectancy. Participants with response times quicker than 10 min or incomplete data (*n* = 167) were excluded prior to analysis because a pretest on the survey items revealed that it took at least 10 min to read the instructions and reply to all items (the study was advertised as taking approximately 30 min to complete). Based on these criteria, the study included 224 Danish men (*M*_age_ = 24.95 years, *SD* = 3.51), with the data collected during the spring semester of 2018 (until early May).

Participants came from two distinct pools (*n*_low reward_ = 143; *n*_high reward_ = 81), in which the first pool received a smaller monetary reward for participating (approximately US$5), whereas the second one received a larger monetary reward (approximately US$22). These two pools of participants did not differ in terms of age or several other demographic measures and, as such, can be assumed to represent two relatively matched groups.

The study was approved by the local ethical review board (Project ID: 0220). Participants from the high monetary reward group were sent an email 1 week before the study, with a consent form and information about which kinds of measurements they would be asked to reply to in the study. Three days later, they got a new email stressing the importance of reading the consent form. Subsequently, on the day of the study, participants received an email with a link to Qualtrics. Upon entering the page, participants read and signed the consent form. Next, they were informed that, in order to complete the study, they needed a ruler or a printer to print the ruler that was attached in the email. Participants from the low monetary reward group were sent directly to the consent form and the information there, as these participants were recruited and monitored through a professional marketing survey agency.

All participants provided written informed consent and the study was conducted in accordance with the World Medical Association Code of Ethics (Declaration of Helsinki) for human experimentation. All participants were paid regardless of whether they provided complete data in all tasks, could withdraw from the study at any time without negative consequences, and had the right to contact the principal investigator to seek more information about the study (no participants did). Moreover, as the study requested participants to measure their erect and flaccid penis size, the consent form emphasized that the penis measurements could cause psychological stress, such as discomfort in the measurement situation and worries about not getting erection. Participants were also ensured that the data were collected anonymously, and that their unique replies in no way could be identified in the reporting of the results.

For the measures related to the present article, participants replied to a set of items linked to bodily markers. Specifically, they indicated their athleticism on a scale ranging from 1 (not athletic) to 9 (very athletic); their height in centimeters, their weight in kilograms; and, crucially, their penis size in flaccid and erect states (in centimeters and followed by careful instructions as how the measurements should be done both in the flaccid and erect state). To be able to reliably take all these measurements, participants were sent a ruler and a guide of how to print it out. The ruler was sent in PDF format (scale 1:1) to make sure no alterations could be made with respect to its format. Further, it was specifically mentioned as part of the instructions that the ruler had to be printed in A4 format. Note that the measures were collected through different response formats, which is a fruitful way to mitigate problems associated with common method bias (Podsakoff et al., 2003; Gasiorowska et al., 2022).

## 3. Results

### 3.1. Outliers and attrition check

As conservative outlier criteria for height and penis size, we excluded all men who either reported being shorter than the threshold of 147 cm for dwarfism (*n* = 2; *cf.* Pritchard, 2021) or who claimed to be taller than the Guinness World Record of 272 cm (*n* = 1; Silk, 2006) and men who reported that their erect or flaccid penis size was either 0 cm (*n* = 1) or larger than the claimed world record of 34 cm (Kimmel et al., 2014; Kim, 2016; Zane, 2021) in the erect (*n* = 12) or flaccid (*n* = 14) state. In the most extreme case, the self-reported erect penis size (9,000 cm) was 50 times larger than the penis size of an adult elephant, which has the largest penis of any land animal (Giustina, 2005). In total, this resulted in the exclusion of 21 participants’ data, as some of the described cases were multiple outliers. Our data did not include any extreme values on weight when the outliers for penis size and height had been excluded. After excluding 12 additional outliers who had the survey active for an unrealistically long time before sending it in (at least 4 h and at most 5 days), the average survey completion time was approximately 22 min (*M* = 21.79 min, *SD* = 16.57).^1^ Based on these criteria, our final sample comprised 191 participants (*M*_age_ = 25.09 years, *SD* = 3.61), with 121 participants in the low monetary reward group and 70 participants in the high monetary reward group. Attrition due to our exclusion criteria of response time and the above-stated physical attributes was not associated with the amount of payment that participants received, as evidenced by a 2 (monetary reward: low, high) × 2 (participant excluded: now, yes) Chi-square analysis [χ^2^(1, *N* = 224) = 0.13, *p* = 0.71, Cramér’s *V* = 0.02].

### 3.2. Main analyses

Similar to other common paradigms, such as coin-flip tasks or die-roll tasks that are frequently used in moral psychology (Gerlach et al., 2019), our main analyses focus on participants’ deviation from a theoretical mean or the scale midpoint, meaning that exaggerations cannot necessarily be detected *individually* but rather at the *group level*. As there is no available Danish mean for flaccid penis size, we only present data pertaining to erect penis size in relation to our first study objective. However, when comparing the participant pools to address our second main objective, we report data on flaccid penis size.

Irrespective of athleticism, height, and penis size, participants’ self-reported measures deviated from the available Danish population mean or the scale midpoint on all these physical attributes, albeit only marginally for athleticism. Indeed, participants reported being marginally more athletic than the scale midpoint of 5 (*M* = 5.21, *SD* = 1.66; *t*(190) = 1.79, *p* = 0.075) and their self-reported height was significantly above than the Danish mean of 180.4 cm (Statistical Yearbook, 2017) for men of similar ages [*M* = 182.19 cm, *SD* = 7.66; *t*(190) = 3.22, *p* = 0.002].^2^ This represents a height deviance of approximately 1% compared to the Danish mean and supports earlier investigations in which men have been shown to exaggerate their height (Toma et al., 2008; Bogaert and McCreary, 2011; Pozzebon et al., 2012).

However, the most extreme deviance from the Danish mean (as reported at WordData.info, n.d.) was found for erect penis size, in which participants’ self-reported size (*M* = 18.02 cm, *SD* = 3.72) was 21.1% larger than the stated Danish mean of 14.88 cm [*t*(190) = 11.67, *p* < 0.001].^3^ Fisher’s *r*-to-*z* transformation revealed that the effect size for the plausible over-reporting in erect penis size relative to the stated Danish population mean (*r* = 0.645, *N* = 191) is significantly greater than the meta-analytic effect size for the positivity bias in attributions (*r* = 0.433, *N* = 41,538; *z* = 4.15, *p* < 0.001), implying that men, on average, exaggerated the size of their erect penis relatively more than people’s general propensity to portray themselves in a self-serving way (Mezulis et al., 2004).

Consistent with former investigations comparing Danes’ self-reported and measured weight (Neermark et al., 2019), we found no significant difference between participants’ self-reported weight and the available Danish mean of 81.9 kg (*M* = 81.64 kg, *SD* = 15.64; *t*(190) = −0.23, *p* = 0.82), as reported by the State Institute of Public Health (2018). As shown in Table 1, zero-order correlations revealed that erect, but not flaccid, penis size was significantly positively correlated with all other physical attributes, consistent with several previous studies (Siminoski and Bain, 1993; Ponchietti et al., 2001; Lever et al., 2006; Veale et al., 2015).

**Table 1:** Zero-order correlations between erect and flaccid penis size, athleticism, weight, and height.

	Penis size erect	Penis size flaccid	Athleticism	Weight	Height
Penis size erect	1	0.58***	0.17*	0.19**	0.21**
Penis size flaccid	–	1	0.18*	0.05	0.13+
Athleticism	–	–	1	−0.02	0.11
Weight	–	–	–	1	0.32***
Height	–	–	–	–	1

+ *p* < 0.10, * *p* < 0.05, ** *p* < 0.01, *** *p* < 0.001.

Thus, it can be concluded that participants likely exaggerated their level of athleticism, presumably as a self-view-bolstering tactic, and that their height and penis size estimates, but not weight, may have been higher than their true scores on these variables, as participants consistently scored significantly above the Danish mean on both height and penis size; especially (and dramatically) so for penis size. Further, if participants exaggerated their height but not their weight, they effectively portrayed themselves as more physically fit.

With respect to the second aim of exploring whether the amount of monetary rewards influenced men’s response pattern on these self-view-relevant attributes, we found no significant difference between the participant pools in athleticism, height, and weight (*F*s < 1; see Table 2). However, in terms of penis size, there was a statistically significant difference between the participant pools [*F*(1, 189) = 7.01 *p* = 0.009, *η*^2^ = 0.04], in which the group receiving the smaller monetary reward self-reported a greater erect penis size than the group receiving the larger monetary reward (see Table 2 for means and standard deviations). The same pattern of results was found for flaccid penis size [*F*(1, 189) = 5.39, *p* = 0.021, *η*^2^ = 0.03], with the group receiving the smaller monetary reward again self-reporting a greater penis size than the group receiving the larger monetary reward (see Table 2). Interestingly, participants in the low payment group (*M* = 19.59 min, *SD* = 17.03) had a shorter average response time than participants in the high payment group [*M* = 25.60 min, *SD* = 15.11; *F*(1, 189) = 5.99, *p* = 0.015, *η*^2^ = 0.03]. However, the group-penis size link was not mediated by participants’ response time.

**Table 2:** Means (and standard deviations) across participant pools for athleticism, height, weight, and erect and flaccid penis size.

	Low monetary rewards	High monetary rewards	F statistic
Athleticism (1–9)	5.24 (1.63)	5.17 (1.72)	0.08
Height (cm)	182.10 (8.54)	182.33 (5.92)	0.04
Weight (kg)	81.74 (16.16)	81.46 (14.82)	0.01
Erect penis size (cm)	18.55 (4.35)	17.10 (1.96)	7.01**
Flaccid penis size (cm)	12.18 (4.65)	10.78 (2.53)	5.39*

*p < 0.05, **p < 0.01.

### 3.3. Supplementary analyses

A Pearson’s Chi-square analysis on the entire final sample, including the outliers (*N* = 224), revealed that participants receiving the smaller monetary reward were significantly more likely (7.7%) to report that their erect penis size was larger than the claimed, but not confirmed, world record of 34 cm (Kimmel et al., 2014; Kim, 2016; Zane, 2021) compared to participants receiving the larger monetary reward (1.2%; χ^2^(1, *N* = 224) = 4.25, *p* = 0.039, Cramér’s *V* = 0.14]. Similarly, participants receiving the smaller monetary reward were significantly more likely (9.1%) to report that their flaccid penis size was larger than the claimed world record compared to participants receiving the larger monetary reward (1.2%; χ^2^(1, *N* = 224) = 5.45, *p* = 0.020, Cramér’s *V* = 0.16]. We found the Chi-square analyses informative because a self-reported penis size above 34 cm is arguably easier to categorize as a “true lie”—at the *individual* level—compared to our main analyses, which only compare the sample mean with the population mean—at the *aggregate* level.

## 4. Discussion

The present study shows that men seem to self-report their physical attributes in a self-view-bolstering way, although not for weight, consistent with earlier findings (Neermark et al., 2019). Specifically, at the aggregate level, men reported being marginally more athletic compared to the scale midpoint, claimed to be significantly taller compared to the Danish mean for individuals of similar ages, and stated that their erect penis size was several centimeters longer than the available Danish population mean. The finding that participants do not seem to have over-reported their weight but likely exaggerated their height slightly also implies that they sought to present themselves as more physically fit. Together, these results indicate that, when interested in bodily variables important to men’s self-view and identity, such variables should not be done through self-report; especially not if they concern private bodily measures linked to masculinity (i.e., penis size). Indeed, men deviated substantially more in their reporting of private (vs. publicly visible) body measures, as the overall sample mean in erect penis size was at least 21.1% above the Danish population mean, while only 1% above the Danish mean in height among men of similar ages and roughly equal to the population mean in weight.

Interestingly, giving participants a higher (vs. lower) monetary reward reduced the average self-reported estimate of both erect and flaccid penis size, but had no impact on the more publicly visible measures. To underscore the point that participants in the low monetary reward group provided less accurate self-report estimates, we further found participants in this group to be significantly more likely to report that their erect and flaccid penis size was larger than the claimed world record of 34 cm (Kimmel et al., 2014; Kim, 2016; Zane, 2021). However, the means of erect penis size were still significantly above the available Danish population mean for both the low and high payment groups. As such, even with the higher monetary reward, our results regarding private self-report data do not appear to be trustworthy.

While our results indicate that men may have exaggerated their penis size and, to a lesser extent, their height and athleticism in a self-view-bolstering way, it is important to note that extreme values based on self-report can be the result not only of deliberate exaggerations but also of measurement error. We find a measurement error account unlikely to be the main driver of our results for several reasons. First, regarding penis size, the deviation of more than 20% (upward) from the stated Danish population mean is too extreme to realistically have occurred simply due to measurement error, and a measurement error account should arguably stipulate both under- and over-reporting, which is not congruent with the current results. Second, self-reported penis size has previously been found to correlate positively with social desirability scores (King et al., 2019), suggesting that some men deliberately exaggerate their penis size. Still, our study would have been strengthened by asking participants to also measure other body parts with the ruler that are not commonly connected to masculinity (e.g., their forearms). Such instructions would have allowed us to more explicitly test whether, as we believe, men strategically exaggerate only those bodily cues that are linked to masculinity or, alternatively, whether they over-report *all* bodily measures, irrespective of their “macho” meaning. It is possible that men, on average, are more inclined to lie about their penis size than their height, weight, or athleticism, considering that the penis is typically concealed and hence easier to lie about without getting caught in everyday interactions, whereas people cannot easily hide their height, weight, and body shape.

In conclusion, our results suggest that private data related to bodily cues of masculinity can only be reliably collected in the lab, where conditions can be fully controlled. Given our findings, scientific studies with self-report data concerning penis size should be interpreted with great caution. However, one remedy to reduce exaggerated response patterns seems to be higher monetary rewards given to participants. Indeed, one study found monetary incentives to be the top priority for online panel participants, and further revealed that data quality can be positively related to monetary compensation (Litman et al., 2015), supporting our argument that increased payments may be important for accessing high-quality data on the private (penis) measures investigated herein. It is possible that participants who received the larger monetary payment, on average, were less inclined to exaggerate the size of their penis because they felt a stronger need to reply (more) honestly. In contrast, those who received the smaller monetary payment may have been more motivated to exaggerate their penis size due to anger for the low payment coupled with the activation of self-threat when receiving questions about male markers of masculinity. Indeed, self-threat has been shown to magnify the self-serving bias (Campbell and Sedikides, 1999) and participants receiving the low monetary reward might have been more prone to engage in (extreme) protest responses—as our Chi-square analyses indicate—due to psychological reactance following the low payment (MacKenzie and Podsakoff, 2012).

Future research could examine, for instance, whether oath scripts or the implementation of interactive survey techniques, with direct feedback to participants when their responses exceed certain probability thresholds, may reduce exaggerated response patterns in studies with self-report measures (Kemper et al., 2020). Before such studies are conducted, the most telling take-away message based on the current results—regarding the aggregate “believability” in men’s self-reported penis size—is perhaps best captured by a quote from the New York Times bestselling author Darynda Jones: “Never trust a man with a penis.”

## Data availability statement

The raw data supporting the conclusions of this article will be made available by the authors, without undue reservation.

## Ethics statement

The studies involving human participants were reviewed and approved by the local ethical review board (Project ID: 0220). The patients/participants provided their written informed consent to participate in this study.

## Author contributions

JC methodology, investigation, data curation, formal analysis, writing—original draft, and writing—review and editing. TO conceptualization, methodology, formal analysis, writing—original draft, writing—review and editing, and supervision. C-JL writing—review and editing and supervision. All authors contributed to the article and approved the submitted version.

## Conflict of interest

The authors declare that the research was conducted in the absence of any commercial or financial relationships that could be construed as a potential conflict of interest.

## Publisher’s note

All claims expressed in this article are solely those of the authors and do not necessarily represent those of their affiliated organizations, or those of the publisher, the editors and the reviewers. Any product that may be evaluated in this article, or claim that may be made by its manufacturer, is not guaranteed or endorsed by the publisher.

## Footnotes

^1^The nature and significance of the results do not change by including these 12 cases in the main analyses.

^2^As the population mean regarding height is based on the Danish defense recruitment, with most male conscripts being 17 to 20 years old when their height is measured (Ministry of Defence, 2022), we used the Danish height data from 2012, as conscripts aged 17-20 in 2012 should have a mean age close to our sample mean of 25 years in early 2018 when our study was conducted. Using the population mean of 180.82 cm from the first half of 2018 (Ministry of Defence, 2022) does not change the nature or significance of our results.

^3^We cannot attest to the validity of the penis size metrics provided on WorldData.info; however, it is reasonable to assume that, if anything, the country-specific means of penis size available on this website should be an *over-estimation* of the average penis size for men in the stated countries. For example, whereas the Danish mean is 14.88 cm on this site, studies in urology with the penis size measurements taken by medical professionals have found Caucasian men to have an average erect penis size of around 12.9–13.6 cm (Wessells et al., 1996; Veale et al., 2015; King, 2020). This means that our comparison between the average penis size of men in the current study and the Danish average, as available on WorldData.info, should be a conservative rather than liberal test of our theorizing. In fact, our study mean of 18.02 cm for erect penis size is even greater than most former self-report studies on men’s erect penis size, where the mean has been around 15.6–16.8 cm (e.g., Jamison and Gebhard, 1988; Richters et al., 1995; Smith et al., 1998; Bogaert and Hershberger, 1999; King et al., 2019).
